# Multi-Centre Observational Study of Transplacental Transmission of Influenza Antibodies following Vaccination with AS03_A_-Adjuvanted H1N1 2009 Vaccine

**DOI:** 10.1371/journal.pone.0047448

**Published:** 2013-01-23

**Authors:** Richard Puleston, George Bugg, Katja Hoschler, Justin Konje, James Thornton, Iain Stephenson, Puja Myles, Joanne Enstone, Glenda Augustine, Yvette Davis, Maria Zambon, Karl Nicholson, Jonathan Nguyen-Van-Tam

**Affiliations:** 1 Division of Epidemiology and Public Health, School of Community Health Sciences, University of Nottingham, Clinical Sciences Building, City Hospital, Nottingham, United Kingdom; 2 Nottingham University Hospitals National Health Service Trust, Queens Medical Centre, Nottingham, United Kingdom; 3 Health Protection Agency, London, United Kingdom; 4 Reproductive Sciences, Department of Cancer Studies and Molecular Medicine, University of Leicester, Leicester, United Kingdom; 5 Division of Human Development, Faculty of Medicine and Health Sciences, University of Nottingham, Nottingham, United Kingdom; 6 Department of Infection, Immunity and Inflammation, University of Leicester, Leicester, United Kingdom; Centers for Disease Control and Prevention, United States of America

## Abstract

**Introduction:**

Illness and death from influenza increase during pregnancy. In the United Kingdom pregnant women were targeted in a national programme for vaccination during the H1N1 2009–10 pandemic.

**Methods:**

In this study, pregnant women were recruited in labour from November 9, 2009 to March 10, 2010. Pandemic vaccination status was determined. Venous cord blood collected at delivery was evaluated for transplacental transfer of antibodies by measurement of haemagglutination inhibition and microneutralization titres.

**Results:**

Samples were collected from 77 vaccinated and 27 unvaccinated women. Seroprotection (HI titre ≥1∶40) was detected in 58 (75.3%, 95% CI 64.2–84.4) cord blood samples from vaccinated women and 5 (18.5%, 95% CI 6.3–38.1) from unvaccinated women (P<0.0001). There was evidence of transplacental seroprotection 8 days after maternal immunization (77.9%, 95 CI 66.2–87.1), maintained in most cases for at least 16 weeks.

**Discussion:**

Immunization of pregnant women with AS03_A_-adjuvanted vaccine is followed by transplacental transfer of passive immunity at titres consistent with clinical protection in three-quarters of new-born infants. The findings support national and international pandemic H1N1 2009 recommendations for immunization during pregnancy.

## Introduction

Influenza infection during pregnancy is associated with increased rates of severe illness, hospitalization and death. The risks to mother and child increase as pregnancy progresses, both during seasonal and pandemic influenza, including the recent H1N1 2009 pandemic. [1]–[7] Before 2009, few countries other than the USA recommended vaccination of pregnant women against influenza; however the emergent epidemiological profile of pandemic H1N1 2009 virus led the World Health Organization (WHO), the European Centre for Disease Prevention and Control and several countries, including the UK, to target pregnant women as a priority group for pandemic influenza vaccination. [8]–[11] Nonetheless, despite the lack of any evidence that influenza vaccines are unsafe, concern about the use of medicines during pregnancy and lack of knowledge about antenatal vaccination against influenza (among healthcare workers and patients) have hindered vaccine uptake. [12]–[16]

Infants under 6 months of age experience high rates of influenza and have the highest rates of hospitalization of any age group (other than the over 65 s), [17]–[19] however preventative options are limited for this group. Transplacental transmission of maternal antibodies may therefore be valuable, assuming they provide protection against illness and hospitalisation. Previous studies suggest that this should be the case. Natural maternal antibodies against influenza and also those provided by maternal vaccination with plain (non-adjuvanted) trivalent seasonal vaccine can protect infants or decrease illness severity [20]–[25] although the evidence base for the latter is conflicting. [26], [27]

The H1N1 2009 pandemic was the first time when nationally and internationally an adjuvanted monovalent influenza vaccine was advocated during pregnancy. Our aim was to evaluate whether immunization during pregnancy with one 3.75 µg dose of AS03_A_-adjuvanted split-virion inactivated influenza A/California/7/2009 H1N1 vaccine raised transplacental antibody titres to levels consistent with protection. Our study was undertaken during the second wave of the H1N1 2009 pandemic in the UK, so we were able to compare cord blood samples from vaccinated and unvaccinated women.

## Methods

### Study design and participants

From November 2009 to March 2010, we undertook an observational study at three sites in the UK (Queen's Medical Centre, Nottingham; City Hospital, Nottingham; and Leicester Royal Infirmary, Leicester) investigating the transfer of immunity to babies born to women immunized/unimmunized with monovalent AS03_A_ adjuvanted H1N1 2009 vaccine (Pandemrix®: GSK Biologicals.) as part of the UK national pandemic vaccine program. Participants were not vaccinated as part of the study.

Pregnant women normally resident in the East Midlands who presented for delivery beyond the first trimester were eligible for participation. Women were recruited regardless of age, social class, ethnicity, previous pregnancy and childbirth status, past and current medical history (including current medications), ethnicity, mode of delivery and outcome of the pregnancy. The main exclusions were uncertain vaccination status (H1N1 2009 vaccine), being held in legal custody, participation in another clinical study, non-residence in the East Midlands (UK) and clinical situations requiring cord blood. We included vaccinated women regardless of the interval between immunization and delivery.

All participants provided informed consent. The Leicester, Northamptonshire and Rutland Ethics Committee and participating hospitals approved the study.

Potential participants were approached for consent during the admission for delivery. We recorded participants' vaccination status against H1N1 2009 virus and collected basic demographic data, medical, pregnancy and childbirth history and the method of delivery and outcome of the pregnancy. The date of immunization and vaccine batch number were obtained from primary care records. Subjects who received vaccine before the date of delivery were considered ‘vaccinated’ and those who were vaccinated on the day of delivery or afterwards (or never vaccinated) were classified as ‘unvaccinated’.

We took venous cord blood samples at childbirth for antibody titration against NIBRG-121 virus (generated from A/California/7/2009 and A/PR/8/34 strains by reverse genetics). Sera were separated and stored at −20°C until transfer to the Respiratory Virus Unit, Centre for Infections (Health Protection Agency, UK) for serological analysis. Antibodies were titrated by haemagglutination-inhibition (HI) assay with standard methods, as reported previously. [28] Sera were tested at an initial dilution of 1∶8 and were serially diluted to 1∶16384 to establish end-point titres; sera that were negative were assigned a titre of 1∶4. Specimens were tested blind, in duplicate and the geometric mean values were used in analyses. Seroprotection was defined as a titre of 1∶40 or greater. Sera were also titrated by microneutralization using standard methods. [28] Sera were tested at an initial dilution of 1∶10 and were serially diluted to a titre of 1∶320. Sera that were negative were assigned a titre of 1∶5 and those with titres of >1∶320 were assigned a value of 1∶640. Appropriate positive and negative control sera were included in both assays. Geometric mean titres (GMTs) were calculated from the duplicate assay results for each cord sample and collectively for both vaccinated and unvaccinated participants.

### Statistical analysis

The primary endpoint was the proportion of participants with haemagglutination inhibition titres of 1∶40 or greater. This corresponds with a 50% or greater reduction in the risk of contracting an influenza illness in a susceptible adult population, [29], [30] and is one of three immunogenicity criteria required by the (European) Committee for Medicinal Products for Human Use (CHMP) for licensure of seasonal influenza vaccine, [31] and authorization of pandemic vaccines. [32] The HI assay is used extensively in the assessment of immunity to influenza and in vaccine licensing, but does not measure the full repertoire of antibodies that may be important in protection. There are no correlates of protection using the microneutralization (MN) assay, but MN is increasingly being used in the assessment of mock pandemic vaccines. Accordingly a MN antibody titre of 1∶60 was arbitrarily chosen as the secondary endpoint. For the purpose of assessing the tolerability and immunogenicity of seasonal and pandemic vaccines, CHMP recommend groups of at least 50 persons per vaccine and age-group. We assumed that two thirds of presenting women would be vaccinated, that 50% of those vaccinated would attain ‘seroprotection’, i.e., a HI titre of 1∶40 or greater and that natural infection (giving rise to ‘seroprotection’ in cord blood) would occur in 20% of unvaccinated participants. With 89 subjects (59 vaccinated and 30 unvaccinated), the study was designed to detect an overall 30% difference in the ‘seroprotection’ rate at the 5% significance level with 80% power (2-tailed statistics). However, we allowed for at least a 10% dropout rate and therefore planned to recruit more than 100 subjects.

All statistical analyses were performed in Stata (StataCorp Inc., version 11). Baseline characteristics of vaccinated and unvaccinated mothers were summarized using un-paired t- and rank sum tests for normally and non-normally distributed continuous data respectively and χ2 test for categorical data. The GMTs of each sample's duplicate HI and MN tests were transformed into binary immune/non-immune status using cut-off titres of 1∶40 or greater for HI and 1∶60 or greater for MN. GMTs of HI and MN antibodies were compared using two group mean comparison t-test of log10-transformed titres. We compared proportions of participants that achieved ‘seroprotection’ by χ2 test. Exact (Clopper–Pearson) CIs are reported for all proportional endpoints. No formal adjustments for multiple testing were done. We assessed the role of possible confounding covariates on immunity, including the duration of ‘exposure’ (defined as the interval between the first UK case of pandemic H1N1 2009 infection and date of vaccination plus 10 days (notional time to seroconversion) for vaccinees, or the date of childbirth for non-vaccinees), gestational age and birth-weight, by multivariable logistic regression.

### Blinding

Investigators in the maternity units could not be blinded to the vaccination status of participants. Cord blood samples were labelled with each participant's unique code and laboratory staff titrated the specimens without knowledge of vaccination status.

## Results

From Nov 18 2009 to Mar 20 2010, 117 eligible women were approached and 104 were enrolled: 77 participants were vaccinated before the date of delivery with one 3.75 µg dose of AS03_A_-adjuvanted split-virion inactivated influenza A/California/7/2009 H1N1 vaccine and 27 were unvaccinated. Cord blood samples were collected at birth from all participants. The gestational age of the babies ranged from 34 to 42 weeks. The date of vaccination was available for 74 of 77 vaccinees; vaccine was administered a median of 42 days (range, 1–108 days) before delivery. Infants whose mothers were vaccinated were delivered from Nov 25 2009 to Mar 12 2010 and those whose mothers were unvaccinated were delivered from Nov 18 2009 to Jan 26 2010. Baseline demographic characteristics of the two groups were similar (Table 1) [33], although the date of delivery was a median of 9.5 days later for vaccinees than non-vaccinees (p = 0.0013 - Mann Whitney).

**Table 1:** Baseline characteristics of vaccinated and unvaccinated mothers [33].

	Vaccine recipients (n = 77)	Unvaccinated (n = 27))	p
**Maternal age, y**	31 (29–32)	29 (26–31)	0.1413
**Ethnic origin**			
White	67 (89)	25 (93)	0.6253
Other	8 (11)	2 (7)	
**Pregnancy status**			
1	35 (46)	10 (37)	0.4172
2 or more	41 (54)	17 (63)	
**Childbirth status**			
0	45 (59)	16 (59)	0.9965
1 or more	31 (41)	11 (41)	
**No. of co-morbidities**			
0	67 (87)	22 (81)	0.4815
1 or more	10 (13)	5 (19)	
**Previous obstetric problems**			
0	50 (65)	16 (59)	0.5982
1 or more	27 (35)	11 (41)	
**Mode of delivery**			
Normal, assisted, elective or caesarean unspecified	67 (88)	22 (88)	0.9831
Emergency caesarean section	9 (12)	3 (12)	
**Sex of child**			
Male	38 (50)	16 (59)	0.4079
**Birthweight, grams**	3365 (3223–3507)	3600 (3400–3799)	0.0848
**Gestational age, weeks**	39.3 (38.9–39.7)	40.1 (39.5–40.7)	0.0612
**No. of under 5-y-olds at home**			
0	43 (57)	17 (63)	0.6103
1 or more	32 (43)	10 (37)	
**No. of smokers in household**			
0	54 (82)	16 (70)	0.2169
1 or more	12 (18)	7 (30)	

Data are means for: years/birth weight/gestational age (95% confidence interval) or number (%) for all other parameters.

### Haemagglutination inhibition (HI)

**Figure 1:**
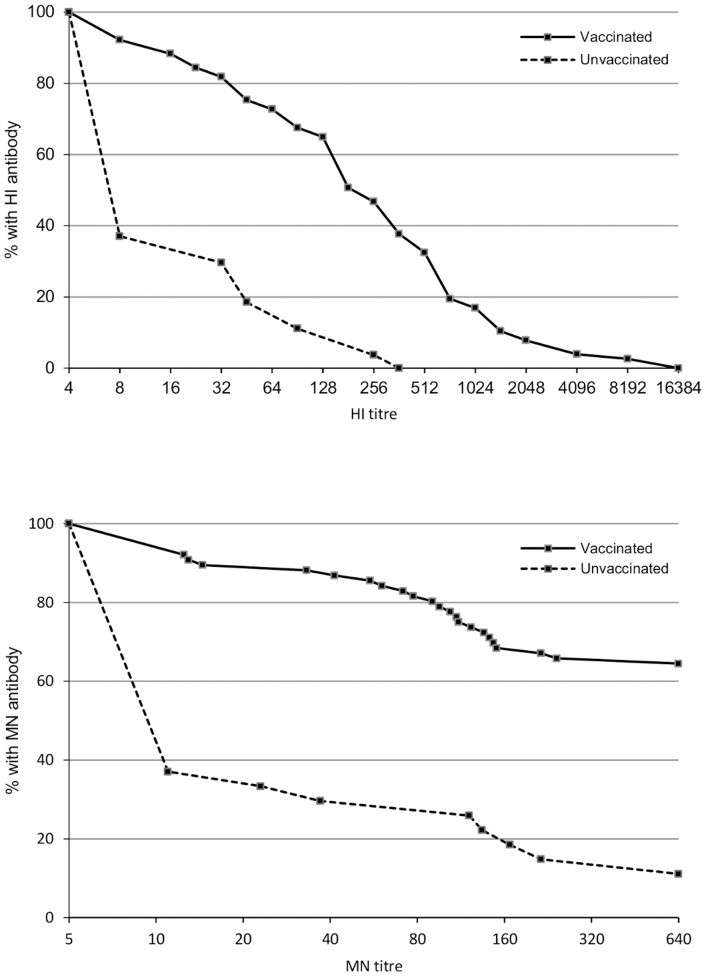
Reverse cumulative distribution curves of haemagglutinination inhibition and microneutralization antibody titres in cord blood serum samples. (Titres are expressed as reciprocal of the dilution and are given on a log_2_ scale.) [33].

**Figure 2:**
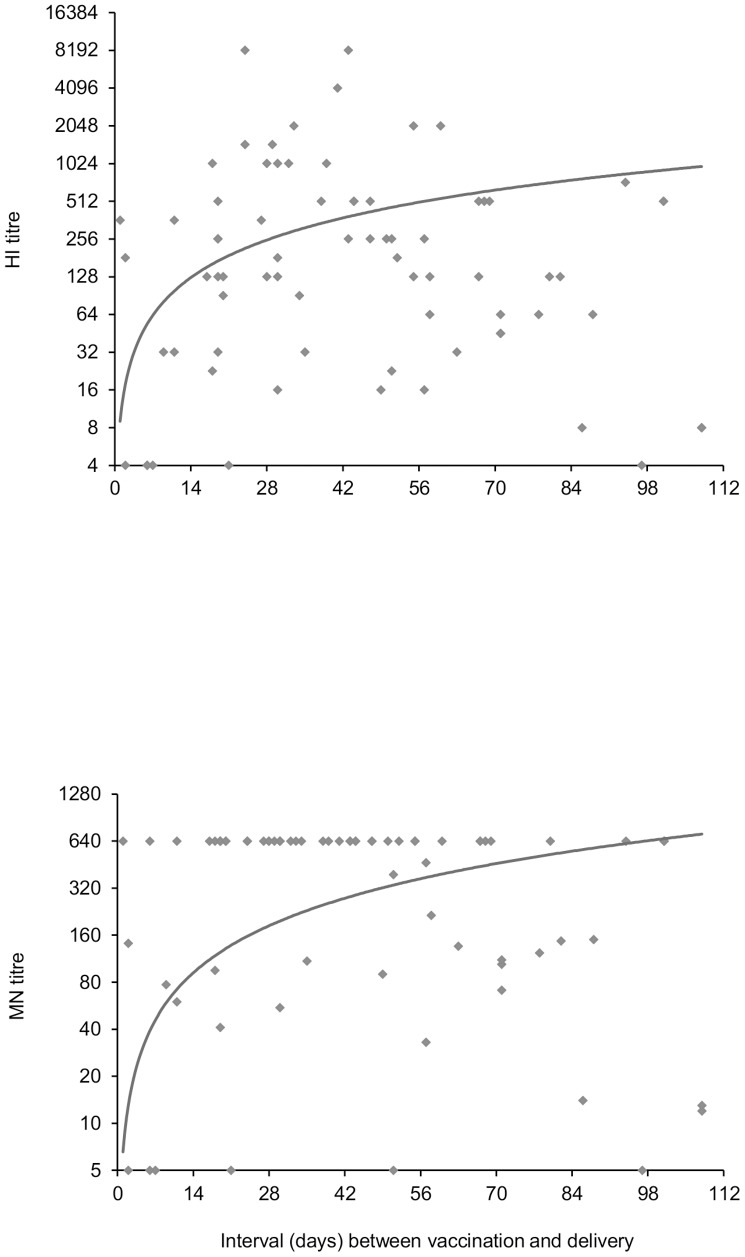
Scatter-plots for cord blood haemagglutinination inhibition and microneutralization titres against interval between vaccination and delivery. (Titres are expressed as reciprocal of the dilution and are given on a log_2_ scale.).

Figure 1 [33] shows the reverse cumulative distribution curves of HI antibody titres in cord bloods from vaccinated and non-vaccinated mothers. HI antibody (titre ≥1∶8) was detected in cord bloods from 10 (37.0%, 95% CI 18.8–55.3) of 27 non-vaccinees and 71 (92.2%, 95% CI 86.2–98.2) of 77 vaccinees (p<0.0001). Levels of HI antibody associated with protection (HI titre ≥1∶40) were found in 5 of 27 (18.5%, 95% CI 6.3–38.1) of non-vaccinees versus 58 of 77 (75.3%, 95% CI 64.2–84.4) vaccinees (p<0.0001). GMTs of HI antibody were substantially higher in cord bloods from vaccinated mothers (148.5, 95% CI 97.4–226.5) than non-vaccinees (9.3, 95% CI 5.6–15.5), respectively (p<0.0001). Among vaccinees, GMTs were comparable in cord bloods from 31 women aged 16 to 29 years (median, 25 years) (126.6, 95% CI 65.2–246.0) and 46 women aged 30 to 44 years (median 34 years) (165.4, 95% CI 94.0–291.0) (p = 0.5701). Figure 2 shows HI antibody titres in cord blood in relation to the interval between vaccination and delivery. HI antibody was present (HI ≥1∶8) in 66 (97.1%, 95% CI 89.8–99.6) of 68 cord blood samples from day 8 onwards and ‘seroprotection’ was found in 53 (77.9%, 95% CI 66.2–87.1). With the intercept set at day 0, the trend-line was consistent with the early occurrence of ‘seroprotection’. The median GMT of HI titres in cord samples taken within one week of vaccination was 4 (inter-quartile range: 4–49) and beyond the first week after vaccination was 181 (59–5120).

### Microneutralization

Figure 1 [33] also shows the reverse cumulative distribution curves of MN antibody titres in cord bloods from vaccinated and non-vaccinated mothers. MN antibody (titre ≥1∶10) was detected in cord bloods from 10 (37.0%, 95% CI 19.4–57.6) of 27 non-vaccinees and 70 (92.1%, 95% CI 83.6–97.0) of 76 vaccinees (p<0.0001). MN titres of ≥1∶60 were found in cord bloods from 7 (25.9%, 95% CI 11.1–46.3) of 27 non-vaccinees versus 64 (84.2%, 95% CI 74.0–91.6) of 76 vaccinees (p<0.0001). GMTs of MN antibody were 12-fold higher in cord bloods from vaccinated mothers (188.3, 95% CI 132.8–267.1) than non-vaccinees (15.5, 95% CI 8.0–29.9) (p<0.0001). Among vaccinees, MN GMTs were comparable in cord bloods from 30 women aged 16 to 29 years (median: 25 years) (214.1, 95% CI 126.0–364.0) and 46 women aged 30 to 44 years (median 34 years) (173.2, 95% CI 107.5–279.0) (p = 0.5042). MN antibody was present at a titre of ≥1∶10 in 64 (95.5%, 95% CI 87.5–99.1) of 67 cord blood samples from day 8 onwards, at titres of ≥1∶60 in 58 (86.6%, 95% CI 76.0–93.7) and at titres of 1∶640 in 45 (67.2%, 95% CI 54.6–78.2). With the intercept set at day 0, the trend-line was consistent with the observations for HI antibody, i.e., early appearance of ‘seroprotection’. Median GMT of MN titres in cord samples taken within one week of vaccination was 74 (inter-quartile range: 5–516) and beyond the first week after vaccination was 640 (117–640).

### Duration

**Figure 3:**
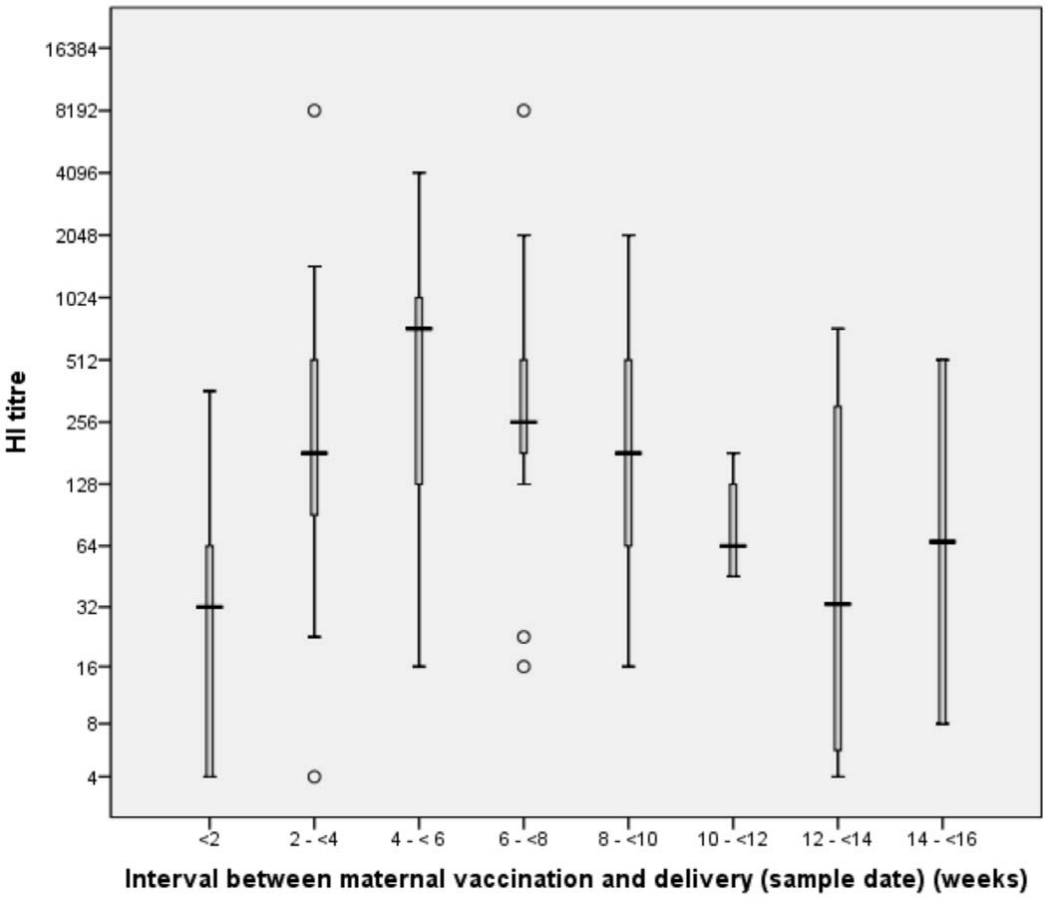
Boxplot GMT haemagglutinination inhibition titre by interval from maternal vaccination to delivery. (Titres are expressed as reciprocal of the dilution and are given on a log_2_ scale.).

Duration of antibody titre protection has also been estimated. Figure 3 shows the median GMT of HI titres against the interval from maternal vaccination to date of delivery (sample date). The median GMT of HI titres peaked 4–6 weeks post vaccination then remained above 1∶40 until the final assessment time-point at 14–16 weeks. The median GMT of MN titres (not plotted – too few data points) peaked earlier at 2–4 weeks post vaccination and remained above 1∶60 to 14–16 weeks. Inter-quartile ranges demonstrate some variability of protective titre duration, the first quartile dropping below both 1∶40 (HI) and 1∶60 (MN) by 12–14 weeks.

The relationship between HI and MN titres is displayed in figure S1.

Examination of potential (*a priori*) confounders by multivariable logistic regression revealed none that reached statistical significance (Table S1) [33].

## Discussion

Our results show that antibodies against H1N1 2009 pandemic virus are present in cord-blood samples from substantially more vaccinated than unvaccinated women and at much higher titres. Our findings are particularly relevant given the substantial impact of H1N1 2009 virus on pregnant women [4]–[7] and the high hospitalization rates and increased morbidity among young infants. [18], [34], [35] However, these results cannot be applied to babies born before 34 weeks gestation as only babies born after this point were recruited. It is probable that the findings would differ for more premature babies as although active antibody (IgG) transfer begins from about 13–16 weeks gestation it does not reach a peak until the last 4 weeks of pregnancy. [36], [37]

In our study over 90% of cord-bloods from vaccinated mothers had HI titres of ≥1∶8 and MN titres of ≥1∶10. These high sero-prevalence rates (>90%) contrast with levels of 37% for the presence of HI and MN antibodies in cord-bloods from non-vaccinated women. This ‘background rate’ of 37% for both HI and MN antibody in cord blood from non-vaccinated mothers is comparable to sero-incidence rates of 10–40% found in venous blood collected across England from people aged 25- to 44-years during October 2009–February 2010. [38] Although the baseline characteristics of vaccinated and unvaccinated women were generally similar, cord-bloods from vaccinated women were collected a median of 9.5 days after those from unvaccinated women. Multivariable logistic regression modelling showed that duration of exposure had no significant effect on cord antibody findings. In the UK, the majority of women enter secondary care for parturition. The three hospitals where participants were recruited provide secondary and tertiary antenatal and intra-partum obstetric care to expectant women resident in the catchment area around them. It is therefore unlikely that any referral/enrolment bias has occurred. In all, we therefore conclude that the substantial difference in sero-prevalence rates (>50%) was the result of maternal vaccination rather than natural infection of vaccinees.

Although there are no established correlates of protection for HI or MN in children and infants, evidence is accumulating that antibodies against seasonal influenza viruses, at levels that are normally associated with protection, can be found in a high proportion of cord blood samples [39] and neonates [40] following immunization during pregnancy. Our study found that three-quarters of vaccinated women had ‘seroprotective’ cord blood HI antibody titres of ≥1∶40 that are associated with at least 50% protection against symptomatic influenza in adults. [29], [30] The proportion with MN titres above 1∶≥60 were higher than the proportion achieving protective HI titres (84.2% versus 75.3%). This pattern is consistent with the findings of Veguilla et al [41], who found that MN titres were more sensitive but HI titres more specific (for natural infection). Correlates of protection for MN titres are less clear than for HI titres and therefore the choice of cut-off for MN titres was arbitrarily chosen as ≥1∶60. Other studies have used thresholds ranging from 1∶40 to 1∶80 and therefore the midpoint was used in this study [41], [42]. As the primary endpoint was HI titres, and MN titres ≥1∶320 add little further information, endpoint titration for MN analysis was not performed. The MN titres in babies of vaccinated mothers were 12 fold higher than in those from unvaccinated mothers. This may however be an underestimate because endpoint titration MN titres were not available.

There is evidence that maternal immunization with seasonal influenza vaccine prevents laboratory-confirmed influenza in infants, [22], [24] and hospitalizations, [24], [25] so our finding of HI and MN antibodies in cord-bloods from vaccinated mothers, particularly ‘seroprotective’ HI titres of ≥1∶40, is likely to be beneficial during the first few months of life when influenza attack rates and illness severity are high and preventative and therapeutic options for infants are limited.

A key finding in our study was the appearance of antibody in cord blood shortly after maternal immunization. By day 8, 97.1% and 95.5% of cord bloods respectively contained HI and MN antibody against pandemic H1N1 2009 virus, 77.9% of cord bloods had ‘seroprotective’ HI titres of ≥1∶40 and 86.6% had MN titres of ≥1∶60. The trend-line fitted to the scatter-chart of HI antibody titres in relation to time between vaccination and delivery (Figure 2) was consistent with passive transfer of protective levels of antibody by day 7 after maternal immunization. Our cord blood antibody results are consistent with those seen in venous blood one week after immunization of young adults and healthcare workers with AS03_A_-adjuvanted H1N1 2009 vaccine. [28], [43] Our study suggests that vaccination of pregnant women with AS03_A_-adjuvanted H1N1 2009 vaccine as late as one week before the expected date of delivery could benefit both mother and child, even if administered during a localized outbreak. We found no evidence of an age-related decline in either HI or MN antibody levels in pregnant women when assessed in age bands 16 to 29 years and 30 to 44 years. Thus children born to 30 to 44 year-old women seem just as likely to benefit from maternal immunization with AS03_A_-adjuvanted H1N1 2009 vaccine as those born to younger women. It is also evident that vaccination stimulates a sustained maternal response for up to 14–16 weeks; this may have been influenced in part by any natural exposure to antigenically-related influenza virus. (Figure 3). Median titres remain above protective levels to this point, however, it is important to note that the range is wide (possibly because few data points were available at this extreme duration) and that for the first quartile, titres dropped below the protective level by 12–14 weeks. Additional research is needed to elucidate for how long the duration of protection extends. However, the rate of decline of actively induced antibody in mothers may be different to the rate of decline of passively acquired antibody in their infants. Antibody levels in the infant will decline after birth; the precise rate of decline is unclear but may be influenced by organism type, starting levels and the mode of immunity (natural vs. vaccinated) [44]–[49]. Ochola et al demonstrated the half-life of passively acquired maternal RSV antibodies in infants was 79 days. [50] Other studies of the persistence of passive maternal antibodies to H.pylori and measles both suggest that immunity has waned by 6 months after birth [48], [49], [51]; however other work suggests the half-life period for influenza antibodies may vary. [52]–[54]

We found no evidence for an effect of other potential confounders on the observed antibody responses. It should be noted that we only studied cord antibody levels in response to AS03_A_-adjuvanted H1N1 2009 vaccine, so it is unclear whether conventional (i.e., unadjuvanted) split and subunit vaccines, whole virus vaccine, or vaccine with alternative adjuvants trigger H1N1 2009 HI and MN antibody as rapidly in cord blood as in our study, or to the same high titres. (Although two brands of vaccine were available for use in the UK during the pandemic, only the AS03_A_-adjuvanted H1N1 2009 vaccine was recommended for use in pregnant women). [55] Pandemic influenza vaccines containing the MF-59 (oil in water emulsion) adjuvant were not procured in the UK and therefore not available for use in pregnant women. A further novel AF03A (oil in water emulsion) adjuvanted influenza vaccine was given conditional marketing authorisation late in the pandemic (June 2010) for use in Europe, by which time the outbreak was substantially over. The authorisation stated that its use could be considered in pregnancy [56], [57].

### Limitations

This was an ‘emergency’ pandemic research study, designed and executed in a very short time frame and as such there are a number of limitations. Comparison of cord sample antibody titres with maternal serum antibody titres would have provided additional detail; however, maternal serum was not obtained. Older school age children in the household/or those attending a child care facility and previous maternal seasonal influenza vaccination could have affected the results however, these data were not collected and seasonal influenza vaccine was not recommended in the UK for pregnant women until the 2010–11 winter season, immediately post-pandemic. In order to check for recruitment bias it would have been useful to know how many eligible subjects refused consent or declined involvement; however for logistical reasons this information was not available. Ideally the mothers and children in the study would have been followed up by taking serial serological samples to assess the duration of antibody persistence. This was considered, but dismissed as being impractical. The babies have been followed up separately to assess the clinical protective effect of the maternally transferred immunity. This separate element of the study was designed to obtain nasal mucous samples (for determination of the presence of influenza virus PCR) from babies in the original cohort if a respiratory illness occurred in the follow up period. This secondary objective study recently completed and will be reported separately.

This study was not powered to test for safety issues relating to the novel vaccination using an AS03_A_-adjuvanted H1N1 2009 vaccine. Nonetheless no serious adverse events were reported to us during the study.

Our study focused on the presence of HI and MN antibody but did not assess clinical effectiveness. The increased rates of illness and death from pandemic influenza during pregnancy make randomised placebo-controlled trials of the efficacy of immunization during pregnancy with H1N1 2009 vaccine unethical, especially as the WHO and national authorities, including the United Kingdom Departments of Health, recommended vaccination during pregnancy as a priority during 2009–10 (pandemic vaccine) and 2010–11 (seasonal trivalent vaccine). An insight into the protection afforded to mothers and their children by vaccination of pregnant women with H1N1 2009 vaccine could perhaps be obtained from effectiveness studies in countries with large databases that capture all relevant information in primary and secondary healthcare settings. Meanwhile, we consider the decision by the Departments of Health in the UK to target pregnant women for vaccination with an AS03_A_-adjuvanted vaccine was justified from the perspective of the serological protection conferred both to the mothers and their babies. However, we have no equivalent data for the use of non adjuvanted, trivalent seasonal vaccine in pregnancy and so cannot generalize further.

Our work strengthens the need for a better understanding of the relationship between measurable antibody and protective immunity in infancy. Future comparative studies of different vaccines administered during pregnancy to inform vaccine policy are also justified.

## Supporting Information

Table S1 **Multivariate logistic regression model for prediction of immune titre HI ≥1∶40 and MN ≥1∶60 (n = 95)** [**33**] **.**
(DOCX)

Figure S1 **Scatter-plot of haemagglutinination inhibition against microneutralization titres.** (Titres are expressed as reciprocal of the dilution and are given on a log_2_ scale.)
(TIF)
