# Supplementary material for: Multi-Centre Observational Study of Transplacental Transmission of Influenza Antibodies following Vaccination with AS03A-Adjuvanted H1N1 2009 Vaccine
Source: PLoS One. 2013 Jan 23;8(1):e47448. doi: 10.1371/journal.pone.0047448 (PMC3553100; doi:10.1371/journal.pone.0047448)
Supplement: Table S1 — Multivariate logistic regression model for prediction of immune titre HI ≥1∶40 and MN ≥1∶60 (n = 95) [33] . (DOCX) [file pone.0047448.s001.docx]

**Supplementary Table 1, Multivariate logistic regression model for prediction of immune titre HI ≥ 1:40 (n = 96) and MN ≥1:60 (n = 95)**

|  | **HI immune titre ≥ 1:40** |  |  | **MN immune titre ≥ 1:60** |  |  |
| --- | --- | --- | --- | --- | --- | --- |
|  | **Adjusted Odds Ratio*** | **p** | **95% Confidence Interval** | **Adjusted Odds Ratio*** | **p** | **95% Confidence Interval** |
| **Vaccinated** | 21.03 | <0.001 | 5.08 - 87.11 | 45.33 | <0.001 | 8.82 - 232.85 |
| **Duration of exposure (days)^$^** | 1.00 | 0.732 | 0.96 - 1.03 | 0.99 | 0.740 | 0.96 - 1.03 |
| **Gravida (two or more)** | 1.35 | 0.721 | 0.26 - 6.86 | 2.13 | 0.451 | 0.30 – 15.12 |
| **Parity (one or more** | 0.53 | 0.643 | 0.04 - 7.77 | 0.82 | 0.912 | 0.03 – 27.47 |
| **Number of under 5s in household (1 or more)** | 2.35 | 0.446 | 0.26 - 21.07 | 2.98 | 0.480 | 0.14 – 62.17 |
| **Mode of delivery (emergency section)** | 0.92 | 0.916 | 0.21 - 4.04 | 0.46 | 0.357 | 0.09 – 2.40 |
| **Gestational age (≥ 37 weeks)** | 2.10 | 0.430 | 0.33 - 13.16 | 1.24 | 0.870 | 0.10 – 15.87 |
| **Any Past Medical History (one or more)** | 0.62 | 0.528 | 0.14 - 2.72 | 0.97 | 0.973 | 0.16 – 5.84 |
| **Baby weight (g)** | 1.00 | 0.315 | 1.00 – 1.00 | 1.00 | 0.169 | 1.00 – 1.00 |
| **Maternal age at delivery (years)** | 1.00 | 0.914 | 0.92 – 1.10 | 0.94 | 0.354 | 0.83 – 1.07 |

* Each variable adjusted for all others in the final model

^$^ Date since first exposure in the UK (26 April 2009) to either delivery or estimated seroconversion date in

unvaccinated and vaccinated subjects respectively.
